# Photoacoustic and fluorescence dual-modality imaging of cerebral biomarkers in Alzheimer’s disease rodent model

**DOI:** 10.1117/1.JBO.29.12.126002

**Published:** 2024-12-23

**Authors:** Tianqu Zhai, Wei Zhang, Chenshuo Ma, Yanhui Ma, Yannis Mantas Paulus, Enming Joseph Su, Geoffrey Murphy, Daniel A. Lawrence, Xueding Wang

**Affiliations:** a University of Michigan, Department of Biomedical Engineering, Ann Arbor, Michigan, United States; b University of Michigan, Department of Electrical Engineering and Computer Sciences, Ann Arbor, Michigan, United States; c The Ohio State University, Department of Ophthalmology and Visual Sciences, Columbus, Ohio, United States; d University of Michigan Medical School, Department of Ophthalmology and Visual Sciences, Ann Arbor, Michigan, United States; e University of Michigan Medical School, Department of Molecular and Integrative Physiology, Ann Arbor, Michigan, United States; f University of Michigan Medical School, Division of Cardiovascular Medicine, Department of Internal Medicine, Ann Arbor, Michigan, United States; g University of Michigan, Department of Radiology, Ann Arbor, Michigan, United States

**Keywords:** photoacoustics microscopy, fluorescence imaging, beta-amyloid, neurovascular imaging, Alzheimer’s disease

## Abstract

**Significance:**

Alzheimer’s disease (AD) is a predominant form of dementia that can lead to a decline in the quality of life and mortality. The understanding of the pathological changes requires monitoring of multiple cerebral biomarkers simultaneously with high resolution. Photoacoustic microscopy resolves single capillaries, allowing investigations into the most affected types of vessels. Combined with confocal fluorescence microscopy, the relationship between plaque deposition and small vessel pathology could be better understood.

**Aim:**

We aim to introduce a dual-modality imaging system combining photoacoustic microscopy (PAM) and confocal fluorescence microscopy (CFM) to provide a comprehensive view of both cerebral cortical vessels and amyloid-β (Aβ) plaque in AD mouse model *in vivo* and to identify the pathological changes of these two biomarkers.

**Approach:**

We developed a dual-modality imaging system to image both cerebral vessel structure and Aβ plaque on groups of mice with different ages and phenotypes. Vessel imaging is enabled by PAM, whereas Aβ plaque is imaged by CFM with the aid of fluorescent dye.

**Results:**

The small vessel density in the AD group was significantly lower than in the control group, whereas the Aβ plaque density in the AD group was not only higher but also increased with age.

**Conclusions:**

This dual-modality system provides a powerful platform for biomarker monitoring of AD expressing multi-dimensional pathological changes.

## 1 Introduction

Alzheimer’s disease (AD), a predominant form of dementia, is marked by the presence of amyloid-β (Aβ) extracellular plaques and neurofibrillary tau tangles in the brain.^1^ Representing 60% to 80% of dementia cases, AD is one of the leading causes of mortality in the United States, affecting ∼6.7 million people aged 65 or older age in 2023.^2^ Despite the absence of effective treatment for AD, the detection and quantification of biomarkers play a pivotal role in the staging and early intervention of the disease, as pathological alterations can precede the clinical manifestations of cognitive decline by several years.^3^ In 2018, the National Institute on Aging and Alzheimer’s Association suggested the inclusion of Aβ, pathologic tau, and neurodegeneration in the pathological assessment and staging of AD for research purposes.^4^ Meanwhile, small vessel disease has been closely linked to AD, suggesting that cerebrovascular dysfunction should also be incorporated as a biomarker for AD assessment.^5^ Although numerous biomarkers were found to correlate with AD, the underlying causal relationship and mechanism remain poorly understood. To gain a comprehensive understanding of the disease, imaging techniques capable of providing diverse biomarker profiles are essential.

The state-of-the-art imaging techniques for AD biomarkers vary based on specific targets and objectives, ranging from early-stage diagnosis to fundamental research of pathology. The Food and Drug Administration (FDA) approved positron emission tomography (PET) has been widely used in clinical applications for Aβ and tau deposition imaging.^6^^,^^7^ With the administration of plaque labeling radiotracer (e.g., F18 radioligands), PET facilitates whole-brain imaging diagnostics of AD in humans, albeit the relatively low resolution (∼5  mm) that limits detailed pathological analysis at single-plaque level. Magnetic resonance imaging (MRI), another widely used technique in clinical AD assessment, employs structural visualization of brain atrophy, serving as a crucial metric for gauging disease progression.^8^ Furthermore, advanced functional MRI techniques, including arterial spin labeling MRI (ASL-MRI) and blood-oxygen-level-dependent (BOLD) MRI, elucidate cerebral perfusion and metabolism by offering voxel-wise whole brain blood flow imaging^9^ and quantification of the relative changes of oxygenated hemoglobin (${\mathrm{HbO}}_{2}$) and deoxygenated hemoglobin (HbR).^10^ Although both PET and MRI have been implemented in clinics, their large size, high cost, and relatively low resolution constrain their utility in fundamental research into AD mechanisms.

Recently advancements in optical imaging techniques with high resolution have significantly enhanced AD-related studies. Single-photon or two-photon fluorescence microscopy is typically used for Aβ and tau imaging with administration of fluorescent dye, offering sub-micrometer resolution and enabling detailed observation of the growth of individual plaque.^11^^–^^13^ However, the penetration depth of this method is limited to less than 1 mm due to the scattering of visible to near-infrared light, restricting its application to small-animal models. Photoacoustic imaging (PAI) is a rapidly evolving technology that can be used for brain imaging and AD research.^14^ By illuminating the target with short light pulses, PAI detects the acoustic pulse emission due to the rapid thermal expansion induced by the light absorption. Several approaches have been explored for detecting AD-related biomarkers using PAI. One of the major implementations, photoacoustic tomography (PAT), has been used to image the entire mouse brain for biomarkers such as Aβ and tau deposits^15^^–^^17^ and inflammation,^18^ though its resolution is insufficient for identifying individual plaques and examine the microscopic level of details. The other major implementation, photoacoustic microscopy (PAM), was less utilized in the field of AD. Hu et al. demonstrated the use of PAM for *in vivo* imaging of Aβ plaques in the Congo red-stained mouse brain,^19^ with resolution capable of resolving a single plaque (5  μm), albeit at a reduced imaging depth (∼500  μm) restricting the imaging to the cerebral cortex. PAM has gained particular recognition for its application in cerebral vasculature imaging, offering high resolution (1 to 10  μm) and wide field-of-view imaging of the rodent brain.^20^^–^^23^ Label-free PAM imaging of vasculature relies on the high optical absorption contrast between hemoglobin and other brain tissue. To date, limited studies have explored AD vasculature using PAM, and Guo et al. demonstrated vasculature morphology and size distribution change in live AD mice with PAM.^23^ This study utilized an intact-skull approach which, although having the advantages of reducing complications involved in craniotomy, was prone to light scattering and sound attenuation that could limit the sensitivity especially to capillaries. Beyond vasculature visualization, functional PAM can further provide imaging of blood oxygenation, which relies on the optical absorption difference between ${\mathrm{HbO}}_{2}$ and HbR,^24^ and blood flow speed, which relies on fast acquisition speed to capture the signal variation caused by red blood cell flow.^25^ These capabilities, with the resolution of single capillaries, may offer profound insights into the cerebral pathology in AD.

In this study, we aim to introduce a platform imaging technology as well as the methodology for monitoring multiple *in vivo* biomarkers in AD mouse models. Although several studies^17^^,^^18^ have utilized PAI and fluorescence imaging to characterize AD *in vivo*, the exploration of vasculature at the microscopic level was limited. Our approach not only implements PAM with capillary-level sensitivity but also, by combining CFM, simplifies the procedure of *in vivo*, open skull monitoring of multiple biomarkers at a microscopic regime. The proposed dual-modality imaging system combining confocal fluorescence microscopy (CFM) and PAM is an upgrade of the system described in our previous publication.^26^^,^^27^ The optics were optimized for mouse brain imaging, and the fluorescence modality was upgraded with a confocal configuration. We used CFM for Aβ imaging and PAM for cerebral vasculature imaging on Aβ expressing transgenic mice of different ages. Our PAM modality provides resolution and sensitivity that can visualize a single capillary, and our CFM modality can resolve a single plaque. We performed quantitative analysis on these biomarkers and demonstrated age-related changes of these biomarkers in the AD mice. The purpose of the presented study is not to provide new insight into the mechanisms of AD but to substantiate the utilization of the integrated dual-modality imaging system in AD biomarker detection.

## 2 Methods

### 2.1 Imaging System

**Fig. 1:**
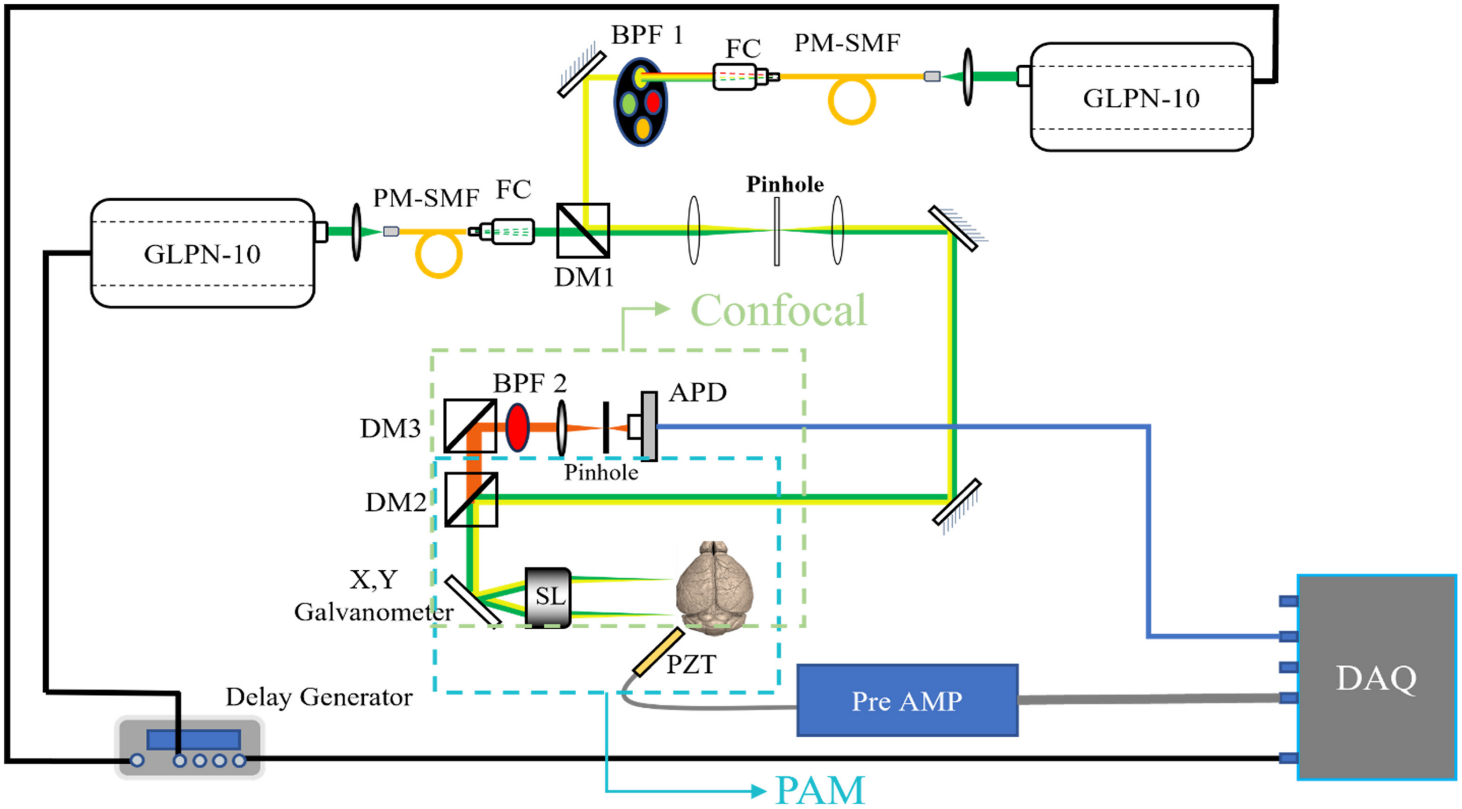
Schematic of the dual-modality imaging system. BPF, bandpass filter; FC, fiber coupler; PM-SMF, polarization-maintaining single mode fiber; DM, dichroic mirror; APD, avalanche photodiode; SL, scanning lens; PZT, piezoelectric transducer; PAM, photoacoustic microscopy.

The imaging setup for the PAM and CFM dual-modality imaging system is shown in Fig. 1. The system employed two pulsed nanosecond fiber lasers (GLPN-16-1-10-M, IPG Photonics) as the illumination source for PAM and CFM. These lasers work at the wavelength of 532 nm, with a pulse duration of 1.5 ns and a pulse repetition rate of up to 600 kHz. Both laser outputs were coupled into polarization maintaining single-mode fibers (PM460-HP, Thorlabs, Newton, United States) with a lens (AC080-016-A-ML, Thorlabs). One of the fibers was 2.6 m long and was used for Raman scattering to generate longer wavelengths. The output of this fiber was collimated by a fiber collimator (CFC11P-A, Thorlabs) followed by a bandpass filter (BPF1 in Fig. 1, FBH560-10, Thorlabs) to precisely select the second Stokes peak at 558 nm. The other fiber was 20 cm long and was intended to generate the same beam profile as the 558 nm beam without inducing any wavelength shift. The output of the 532 nm beam was coaxially combined with the 558 nm beam by a dichroic mirror (DM1 in Fig. 1, FF535-SDi01-25×36, Semrock, Rochester, United States).

The combined beam passed through a 4f system with a pinhole at the Fourier plane to filter out the stray light. It was then sent to a dichroic mirror (DM2 in Fig. 1, Di03-R660-t3-25×36, Semrock) to separate the excitation light and emission light. The excitation beam was steered by a Galvo scanner and then focused by a scan lens (LSM03/LSM02, Thorlabs) down to 8  μm (full width at half maximum, FWHM); thus, a laser point scan could be performed on the sample placed on the focal plane. The depth of view is calculated to be 400  μm, and the imaging depth is around 200  μm, as can be seen in the depth-encoded PAM image (Fig. 4 in the Supplementary Material). To perform photoacoustic signal acquisition, the sample was placed under a water tank with coupling gel, and a customized unfocused needle-shaped piezoelectric transducer with a central frequency of 30 MHz and bandwidth of 50% was used to collect the acoustic signal. To perform fluorescence acquisition, the same laser beam was used for excitation, and the emission light was collected by the same optics except that it would transmit through DM2. Then, the beam was filtered by a bandpass filter (BPF2 in Fig. 1, FBH700-40, Thorlabs), and focused by an achromatic doublet (AC127-025-A, Thorlabs) onto a 20  μm pinhole, and eventually captured by an avalanche photodiode.

The detected photoacoustic signal was amplified by a 70 dB amplification circuit. Both the photoacoustic signal and fluorescence signal were digitized by a DAQ card (RazorMax PCIe CSE161G4, Dynamic Signal Inc., San Bruno, United States) with a 250-MHz sampling rate. A four-channel delay generator (DG535, Stanford Research Systems, Sunnyvale, United States) triggered by the internal clock with a 50 kHz repetition rate was used to simultaneously trigger the GLPN laser, the galvanometer, and the DAQ card. With a scanning area of 512×512 points, it took about 1.75 s to obtain the image. The trigger control and data acquisition were programmed in MATLAB (The MathWorks, Inc., Natick, United States) in coordination with the library of OCTP-1300 scanning head and DAQ card.

### 2.2 Animal Preparation

All the animal procedures were performed in accordance with the National Institutes of Health guidelines and reviewed and approved by the Institutional Animal Care and Use Committee (IACUC) of the University of Michigan (Protocol No.: PRO00010905; PRO00010436). All mice used in this study were bred in-house. The “5× FAD” line (B6.Cg-Tg(APPSwFlLon, PSEN1*M146L*L286V)6799Vas/Mmjax; Strain #034848-JAX) was originally obtained from the Mutant Mouse Resource & Research Centers (MMRRC) and has been maintained on a C57BL/6Tac background since 2012 (<20 generations). As a widely used, commercially available, and well-characterized AD model, 5× FAD had shown both the aggregation of Aβ as well as a decline of vessel density and thus was chosen for the initial validation of our dual-modality imaging system. Twenty-three mice were used in this study, including fourteen 5× FAD (AD) mice and nine age-matched wild-type (WT) mice. The mice were further divided into two age groups, and five AD mice and five WT mice were at 7 to 9 months of age, whereas nine AD mice and four WT mice were at 10 to 12 months of age. To prepare each animal for brain imaging, a cranial window was created. The mouse was first anesthetized by 5% isoflurane mixed in oxygen and kept under 1% to 4% isoflurane gas. A stereotaxic instrument was used to secure the mouse’s head, then the scalp was removed to expose the frontal and parietal skull. A specific 5 mm diameter circular area on the left parietal lobe was chosen for skull removal. The thinning of the skull’s periphery was performed with a dental drill with 0.5 mm diameter (19007-05, Fine Science Tools), and this piece of skull was then removed with sharp forceps. Details of the skull removal were described by Holtmaat et al.^28^ A polyvinyl film (i.e., plastic kitchen wrap) was placed directly on the brain tissue to keep it hydrated, after which the mouse was taken for imaging.

### 2.3 Imaging Procedure

The PAM images were acquired at 532 nm with a diffraction-limited beam focus of 10  μm and a pulse energy of 200 nJ. For each mouse, an imaging field of 2 by 2 mm was captured, comprising 512 by 512 points. For acoustic wave coupling, a water tank with a 1 cm depth and a plastic film base was positioned beyond the imaging area. Ultrasound coupling gel was applied between the water tank and the brain surface to facilitate acoustic wave transmission. A needle transducer was placed inside the water tank and ∼8   mm away from the target region to ensure a homogenous sensitivity could be achieved within the field of view. In this study, PAM images from all 23 mice were used for vasculature imaging and analysis.

Fluorescence imaging was conducted with the administration of CRANAD-3, an Aβ-targeting fluorescence dye (CRANAD-3, Sigma Aldrich, St. Louis, United States) that can penetrate the blood-brain barrier.^29^ CRANAD-3 absorbs light from 520 to 670 nm (FWHM), with a peak at around 600 nm, and emits light from 600 to 750 nm (FWHM). Upon binding with Aβ, CRANAD-3 has an enhanced fluorescence emission which makes it a sensitive and specific dye for Aβ imaging. We used 100 nJ, 1.5 ns pulses at 558 nm for excitation, and 680 to 720 nm for emission detection. The CRANAD-3 solution, prepared at a concentration of 1 mg/mL in a mixture of 15% DMSO, 15% Cremophor, and 70% PBS, was administered intravenously at a dosage of 4 mg/kg. Imaging was performed at 1-h post-injection. Fluorescence images were used for Aβ deposition analysis. A portion of the mice were excluded from the analysis due to the failure of the fluorescence dye injection, including three AD mice from the older group and one WT mouse from the older group.

### 2.4 Data Analysis

**Fig. 2:**
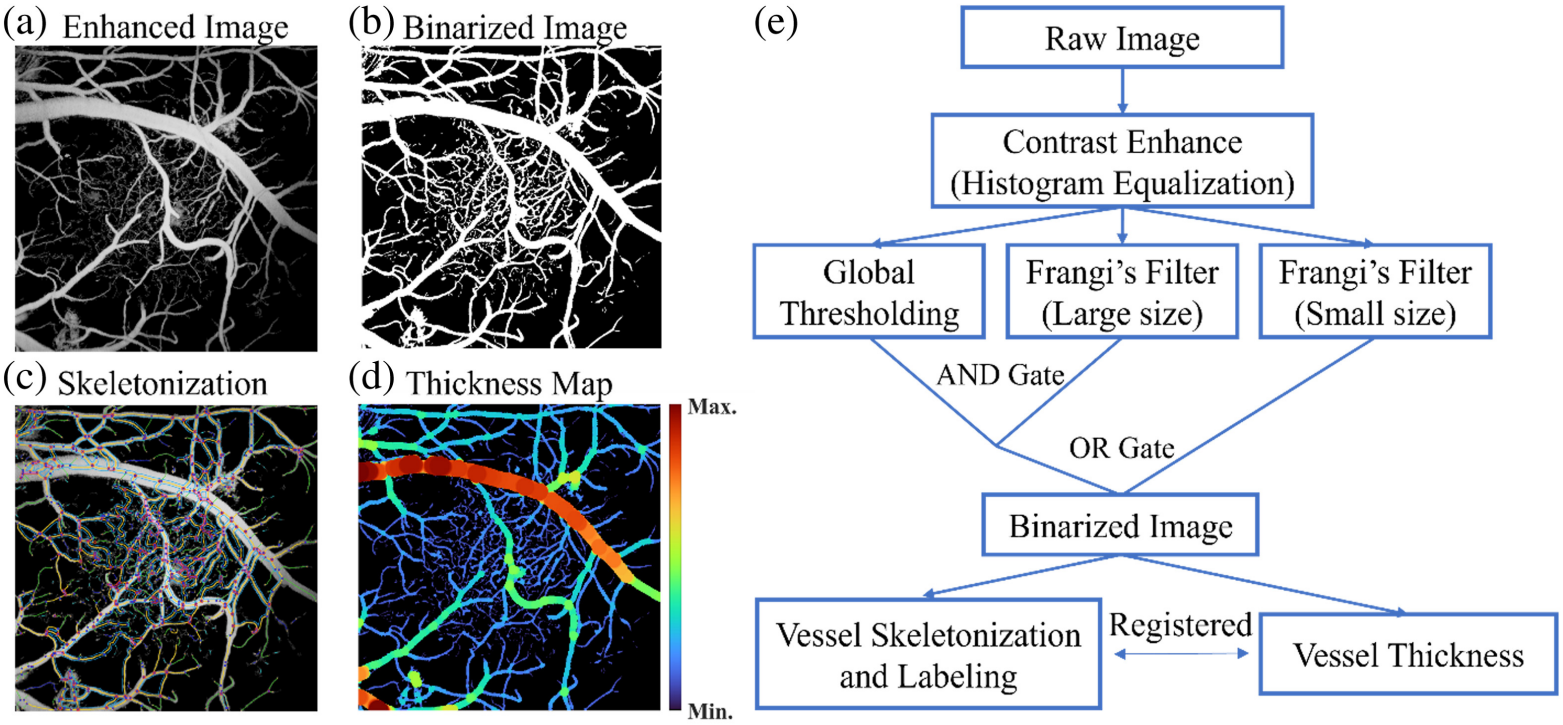
Vasculature metrics derivation methods. (a) An exemplary contrast-enhanced PAM image from a 12-month-old WT mouse. (b) The binarization map of (a) using the proposed method. (c) The skeletonization map overlayed with the PAM image. (d) The calculated vessel size map. (e) The workflow of the vasculature metrics derivation.

Due to the high optical contrast between hemoglobin and brain tissue at 532 nm, the photoacoustic signal amplitude directly reflects the presence of blood vessels. To improve the interpretability of the PAM images, the raw images were enhanced through histogram equalization, as shown in Fig. 2(a), and then analyzed using a modified version of an open-source toolbox, OCTAVA.^30^ The workflow is shown in Fig. 2(e). This modification was particularly focused on refining the implementation of Frangi’s filter within the toolbox, tailoring it specifically for brain imaging applications. The Frangi filter leverages Hessian matrix analysis to enhance the visibility of blood vessels by reducing the impact of intensity variations along vessel structures and suppressing background noise.^31^ The adaptation was aimed at accommodating the wide range of vessel diameters in the brain tissue, from a few microns to over a hundred micrometers. Specifically, we created the binarization map in three different ways:

1. Global thresholding (*MAP1*): A uniform threshold is applied across the entire image to distinguish between vessel and non-vessel pixels. For all the images involved in the analysis, the threshold was set to be 1.1 times of the average pixel value of the background noise. This method largely reflects the true vessel distribution [Fig. 1(a) in the Supplementary Material]; however, it is susceptible to background noise or signal intensity fluctuations, as well as hemorrhage-induced contamination of the images.
2. Frangi’s filter with the small-scale kernel (*MAP2*): This method applies Frangi’s filter with a kernel scale aligned with the size of the smallest vessel, which, in our case, was set to be three pixels (∼4  μm). This method assigns pixel-wise possibility of whether a pixel belongs to a vessel based on the similarity of the surrounding structure to a vessel. It removes background noise as well as hemorrhage signals, promoting the smoothness and connectivity of vessels. However, due to the predetermined kernel size, large vessels may appear fragmented in the center [Fig. 1(b) in the Supplementary Material].
3. Frangi’s filter with the large-scale kernel (*MAP3*): This method applies Frangi’s filter with a kernel scale aligned with the size of the largest vessel, which, in our case, was set to 30 pixels (∼40  μm). This map preserves the integrity of large vessels but may cause small vessels to appear dilated [Fig. 1(c) in the Supplementary Material].

To reconcile the limitations of each map and leverage their benefits, we combined all the three binarization maps using the following equation:

$${\mathrm{MAP}}_{\mathrm{ult}}=(\mathrm{MAP}1\wedge \mathrm{MAP}3)\vee \mathrm{MAP}2.$$

The final binarization map ${\mathrm{MAP}}_{\mathrm{ult}}$, as shown in Fig. 2(b), is then sent to the remaining workflow in the OCTAVA for further analysis. In this study, we analyzed the vessel size distribution and compared it between AD mice and WT mice using an unpaired Student’s t-test.

The Aβ deposition analysis was facilitated by CFM modality with the assistance of CRANAD-3. Each pixel value represents the peak amplitude of a pulsed emission. Due to the sufficient resolution to resolve single plaque deposits, we manually counted the number of plaques in the given $2\times 2\;{\mathrm{mm}}^{2}$ area to represent the severity of Aβ deposition. Comparisons between the AD and WT groups, and within the AD group but between the older age and younger age groups were performed using unpaired Student’s t-test.

## 3 Results

**Fig. 3:**
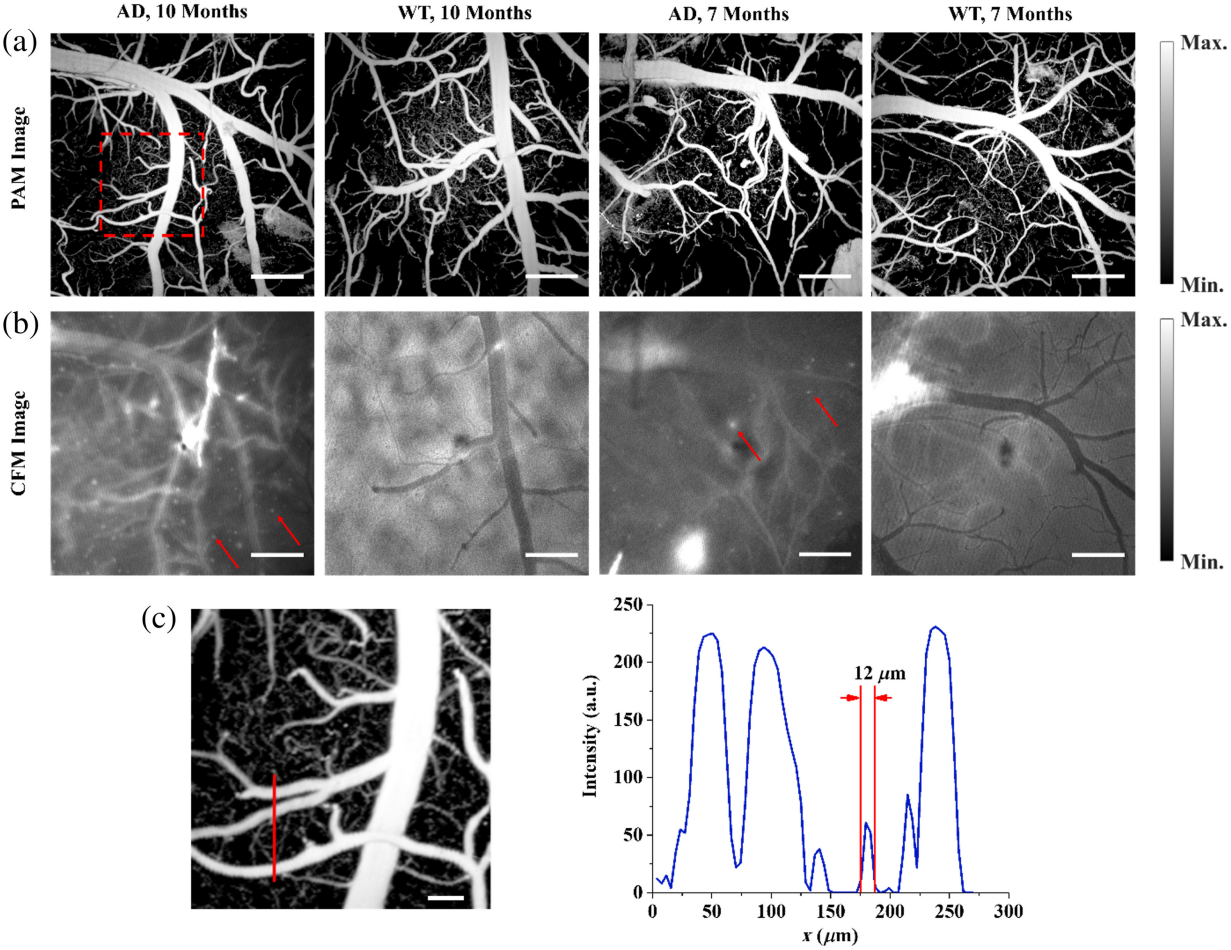
(a) PAM images of four mice, each from a different age group, showing the cerebral cortical vessel structure. The scalebar represents 400  μm. (b) The CFM images from the same cerebral area of the four mice as panel (a), showing the Aβ deposition as labeled by the red arrows. The scalebar represents 400  μm. (c) A zoomed-in section from panel (a) is labeled by the red box. The red line indicates a selected section displayed in (d). The scalebar represents 100  μm. (d) The PA intensity profile of the selected cross-section showing the measured size of different vessels.

Representative PAM and CFM images are shown in Figs. 3(a) and 3(b). The PAM images were used for vasculature analysis and the CFM images were used for Aβ deposition analysis. Here, we presented images from four mice for comparison. From left to right, we have an AD mouse from the older age group (10 months), a WT mouse from the older age group (10 months), an AD mouse from the younger group (7 months), and a WT mouse from the younger group (7 months). Both the PAM images and the CFM images exhibited sufficient quality for vasculature and plaque deposition analyses. In particular, the PAM images have the sensitivity and spatial resolution to detect single capillaries, as indicated by Fig. 3(d), which is the most affected vessel category allowing us to further analyze the AD-associated vascular pathology. Using CFM allows direct identification of Aβ plaques, as shown in Fig. 3(b) labeled by the red arrows. By counting the bright spots in each image, the plaque density was quantified. Since the field of view for each image was the same, the number of bright spots represents the plaque density.

**Fig. 4:**
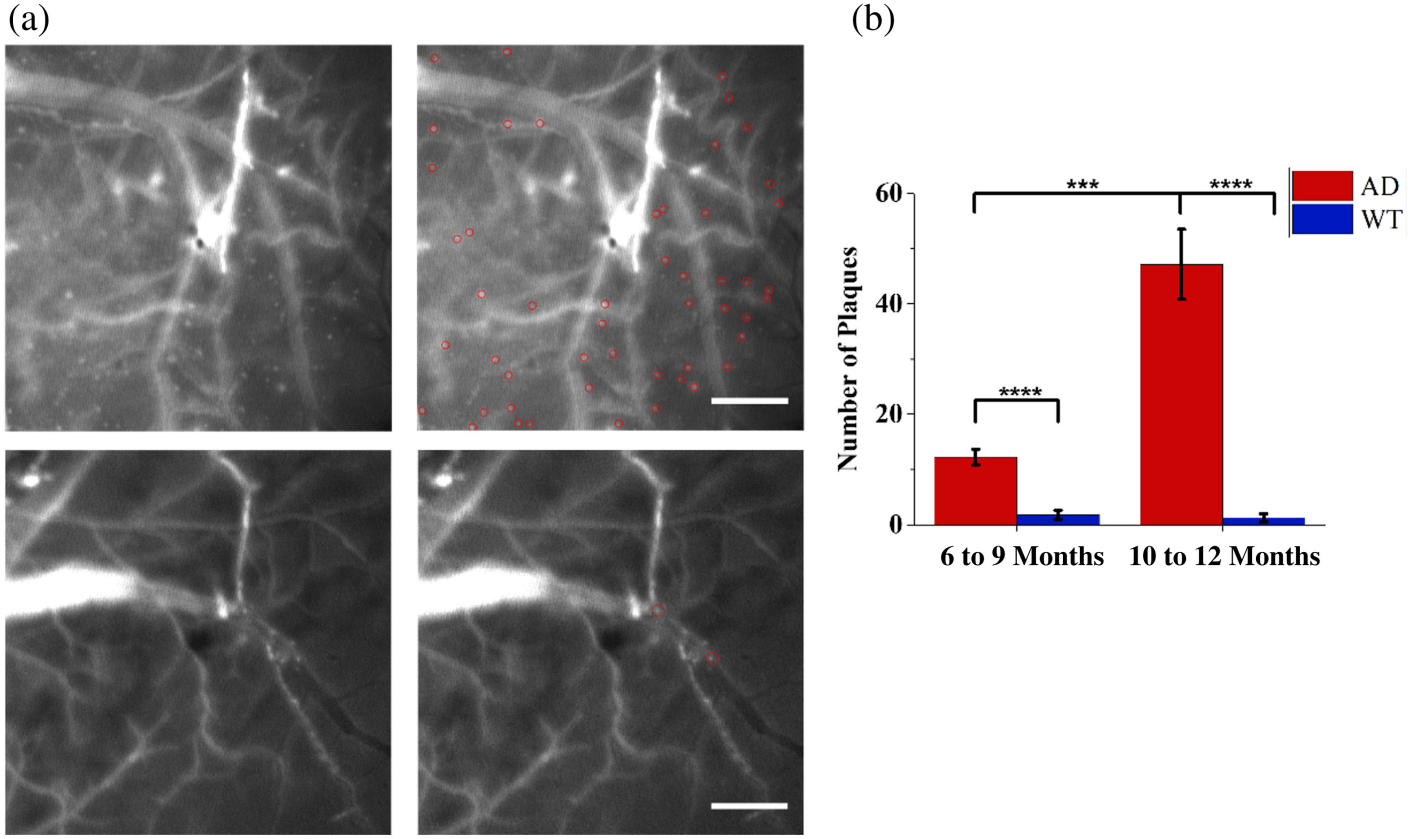
Aβ distribution evaluated with the CFM images. (a) Side-by-side images showing the manual Aβ selection strategy. The top row was from an AD mouse in the older age group, and the bottom row was from a WT mouse in the younger age group. The scalebar represents 400  μm. (b) The comparison in number of plaques between the AD mice and the WT mice for each age group, and between the different age groups for AD mice. The error bar represents the standard deviation. Two-sample *t*-tests were performed for the comparisons. *** indicates p<0.005, and **** indicates p<0.001.

The fluorescence signals were excited with 558 nm light and received through a 680 to 720 nm bandpass filter. Fluorescence images were taken before the injection of CRANAD-3 to verify that no auto-fluorescence signal would be captured by the system (Fig. 2 in the Supplementary Material). After the injection, the dye rapidly perfused through the cerebral vessels, causing a strong background signal across the entire field of view. Gradually, the dye penetrated the blood-brain barrier and diffused into the brain tissue, where most of the Aβ resides. We determined that after 1 h, the contrast between the signals from the Aβ-bound dye and the background signal would be strong enough to identify single plaques. We then analyzed the plaque density by manually counting the number of plaques within the field of view, as shown in Fig. 4(a). We compared the Aβ densities between the AD and the WT groups, and between different age groups of the AD mice, with the results shown in Fig. 4(b). We can clearly see that for both younger and older age groups, the AD mice have significantly higher plaque densities than the WT mice (p=0.00032 for the younger age groups; p=0.00042 for the older age groups). The AD mice in the older age group also exhibit a significant increase in plaque density compared with the AD mice in the younger age group (p=0.0024).

**Fig. 5:**
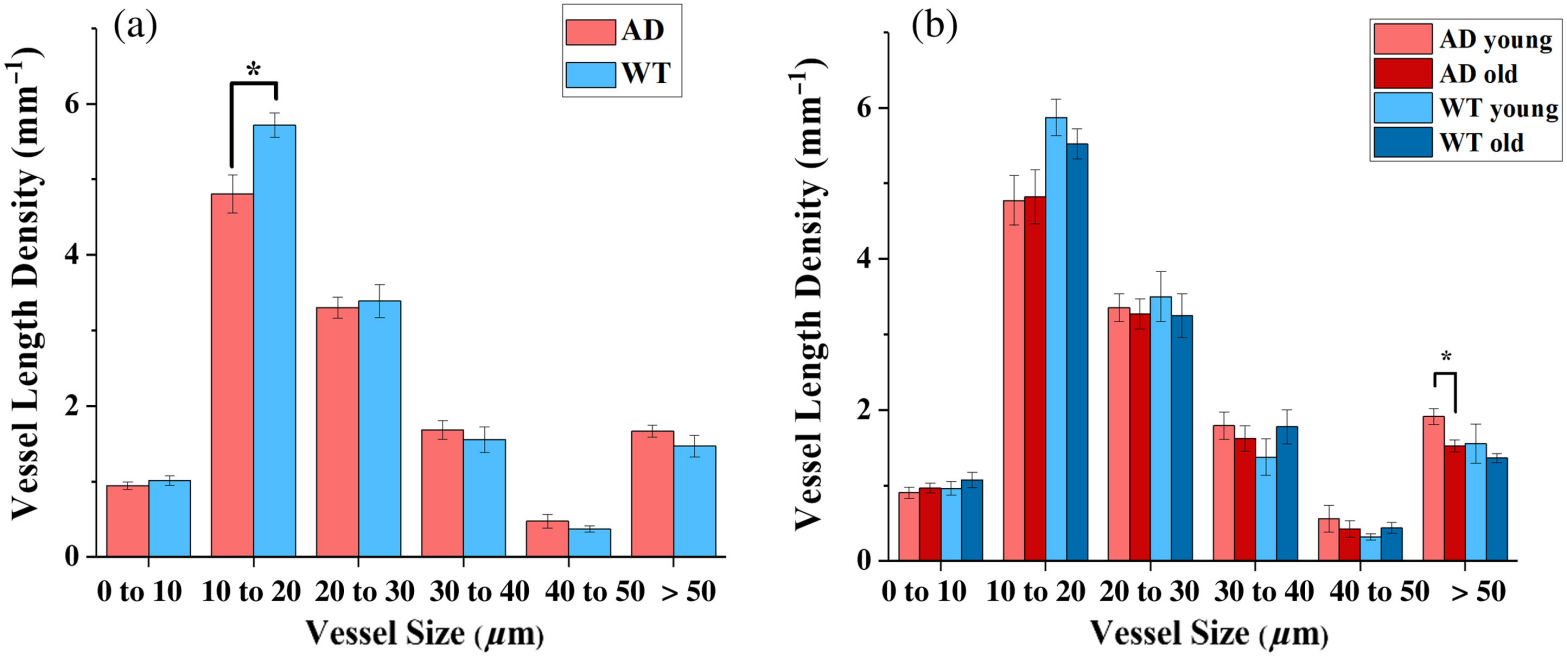
Comparison of vessel length density (VLD) categorized by size. (a) Vessel length density comparison at different size groups between the AD mice and the WT mice. (b) Vessel length density comparison at different size groups between the older and the younger age groups. The error bars represent standard error.

Next, we turn our attention to the vascular analysis empowered by the PAM modality. Unlike Aβ imaging where the difference between the groups is evident from the images, vascular images require quantification metrics for analysis. To perform the morphology analysis, an open-source toolbox, OCTAVA,^30^ was modified, optimized, and used for cerebral vasculature. In Fig. 2(b), we can see that with the modified binarization method (described in the “Methods” section), both the integrity of large vessels and the connectivity and smoothness of the small vessels were captured. From there, we extracted the skeleton of the vessels, as shown in Fig. 2(c), and measured the diameter of each segment of the vessels, as shown in Fig. 2(d).^30^ It should be noted that while Frangi’s filter preserves the integrity of vessels in images that might be compromised by noise, it may also alter the size of vessels due to the pre-determined kernel size. By selecting the same parameters for all the images, the effect of such alteration was consistent for vessels of the same size. Therefore, while the overall size distribution might be shifted slightly, the comparison of the same size group should still be solid. Here, we grouped the vessels by size and compared the vessel length density (VLD), defined as vessel length divided by image area. In Fig. 5(a), we compared the vessel sizes for all the AD mice vs. all the WT mice. We observed that while for vessel sizes larger than 20  μm, there was no significant difference, smaller vessels, especially in the group of 10 to 20  μm, exhibited a significant decrease (p=0.015) in VLD associated with AD. For the vessel sizes in the range of 0 to 10  μm where the VLD was much less, the same trend was observed (i.e., decreased VLD associated with AD when compared with WT), although no statistically significant difference. This observation matched our expectation that AD is associated with the loss of microvessels.^32^

To understand the possible reasons why the VLD in the size range of 0 to 10  μm does not show a statistically significant difference between the AD and the WT groups, we measured the edge spread function (ESF) caused by the finite size of the laser focus (Fig. 3 in the Supplementary Material). We found that the broadening of a sharp edge in our PAM image reached a FWHM of 8  μm. For *in vivo* vasculature measurements, this could lead to a size broadening of 0 to 16  μm. Considering this, we believe that most of the 10 to 20  μm size vessels in our PAM images were from capillaries with sizes <10  μm. In addition, we also compared different age groups within each phenotype, as shown in Fig. 5(b). In the observed age range (7 to 12 months), no significant changes in VLD were observed in all sizes of vessels for WT mice; while for AD mice, the large vessel (>50  μm) density significantly decreased with age (p=0.012).

## 4 Discussion and Conclusion

This study demonstrated the successful *in vivo* detection of multiple biomarkers, distinguishing the AD mice from the WT mice. The proposed dual-modality imaging system provides spatially co-registered images of distinct biomarkers, with each modality delivering high-quality imaging sufficient to detect the pathological alterations caused by AD. This study exemplifies the application of a multi-modality platform we have previously described,^26^^,^^27^ aimed at examining disease models characterized by multiple biomarkers. Enhancements over prior publications include system optimization for brain imaging and improvements in fluorescence imaging via a confocal design. The significance of the confocal design, especially for Aβ imaging in the brain, lies in its ability to exclude undesired light in the axial direction. Given the fluorescence dye’s diffusion throughout the brain, the emission along the axial direction would compromise the contrast of Aβ-bound dye in the horizontal direction. However, the effective sampling of a thin layer of brain tissue enabled by the confocal design allows the identification of individual plaques. Overall, the methodology described above provides a convenient and effective way of investigating neurodegenerative diseases, while simplifying the process to obtain multiple biomarkers. Such a tool could benefit the longitudinal animal studies, which typically involve diseased animals that are aged and fragile, through evaluating treatment effects, investigating disease etiology, and eventually benefiting translational studies for clinical applications.

Quantitative analysis revealed an age-correlated increase in Aβ plaque density within AD-affected brains, aligning with the findings from several previous research on AD mice.^33^^–^^35^ For example, Kawarabayashi et al. observed that insoluble forms of Aβ started to accumulate at 6 to 7 months and exponentially increased from 6 to 10 months in Tg2576 mice.^33^ Bader et al. observed Aβ on the cerebral cortex from 5 months onward until 10 months, in 5xFAD phenotype mice.^35^ These findings match well with our observations, affirming the reliability of our CFM imaging modality.

The analysis of vasculature noted a significant impact on small vessels by AD, which was consistent with a previous study using scanning electron microscopy (SEM).^32^ Although PAM could not reach a resolution comparable to SEM, its *in vivo* applicability offers unique advantages for AD research. Compared with other common *in vivo* angiography techniques, PAM also has unique advantages in terms of AD-related research. Optical coherence tomography angiography (OCTA), as an example, has also been used to study rodent brain vasculature.^36^^,^^37^ OCTA’s detection of vessels relies on the flow of the red blood cells. In the AD brain, however, some of the capillaries may be stalled^38^ and thus could disappear on OCTA. Fluorescence angiography, on the contrary, has also been used in AD-related vasculature research.^39^ Due to the potential interference between Aβ targeting dye and contrast agent needed by fluorescence angiography, we did not choose it for vasculature imaging. PAM relies on the endogenous optical contrast facilitated by hemoglobin to show the presence of blood vessels and thus does not have the aforementioned issues. In addition, PAM has the potential of functional imaging, including blood-oxygenation imaging and flow speed imaging,^20^^–^^22^ which could further empower the research on AD.

It should be noted that in this study, age-related microvascular changes^36^ were not observed. This may be because the span of the age range (7 to 9 months versus 10 to 12 months) was not large enough. In addition, the limited spatial resolution offered by the current system may have caused a misrepresentation of the capillary size. Finally, in the fluorescence results, the background signal from brain tissue and blood vessels interfered with the Aβ signal. Further investigation is needed to understand the optimal imaging time when the background dye is largely metabolized while the dye bound to Aβ still remains.

There are several improvements that can be considered in future studies. First, since both vasculature and Aβ plaques can be imaged *in vivo*, a longitudinal study of the same mouse over several months could be performed. In age-dependent neurodegenerative diseases like AD, such longitudinal monitoring could provide valuable information about disease progression and interactions among multiple biomarkers. Second, the blood oxygenation and blood flow map could be added as additional functional biomarkers, which may provide local perfusion and metabolic information in the brain. There have been studies showing that the resting state blood flow^9^^,^^38^ and the cerebral metabolic rate of oxygen^40^ are compromised in the AD brain. By providing high-resolution information on these functional changes, we can provide a more comprehensive understanding of the mechanism of this disease.

In summary, we have developed a dual-modality imaging system that has the sensitivity to detect the Aβ deposition and the microvascular change in rodent AD models *in vivo*. This platform provides a practical and new tool for longitudinally investigating multiple biomarkers related to AD with high spatial resolution and may contribute to the understanding of the mechanism of AD-induced pathological changes.

## Supplementary Material



## Data Availability

The code and datasets used during the current study are available from the corresponding author on reasonable request.
