# Supplementary material for: Photoacoustic and fluorescence dual-modality imaging of cerebral biomarkers in Alzheimer’s disease rodent model
Source: J Biomed Opt. 2024 Dec 23;29(12):126002. doi: 10.1117/1.JBO.29.12.126002 (PMC11665203; doi:10.1117/1.JBO.29.12.126002)
Supplement: Supplementary file 1 [file JBO_029_126002_SD001.docx]

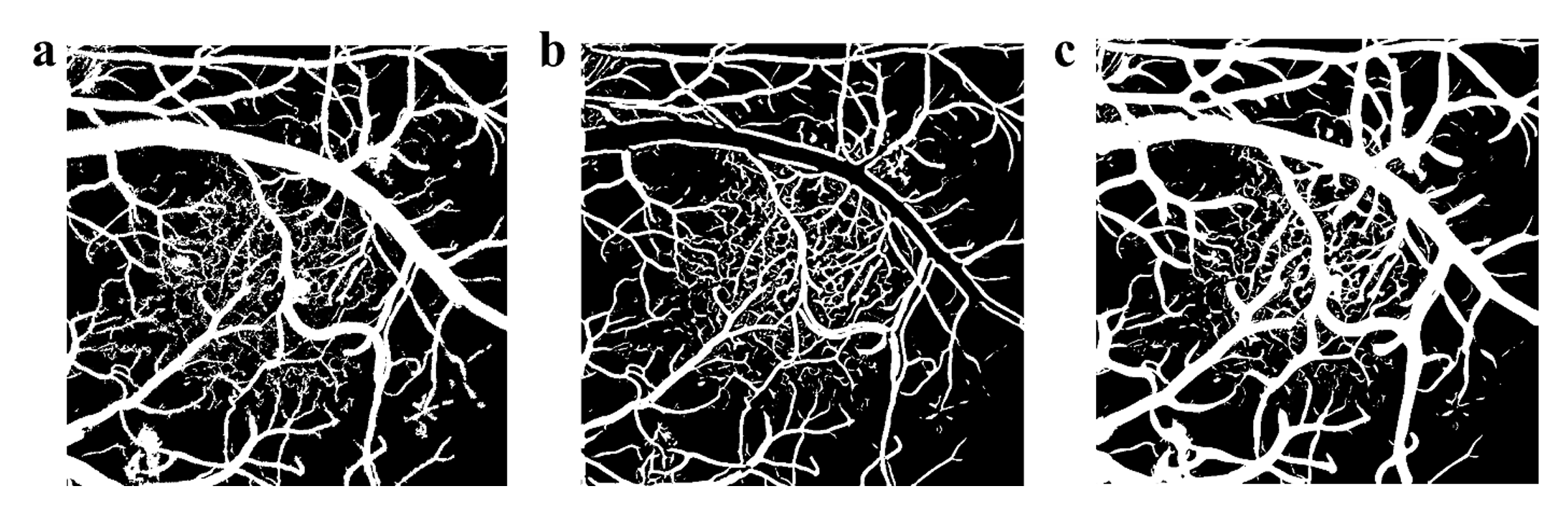


*Supplementary Figure 1. Binarization performance of different methods. (a) Binarization map using global thresholding. (b) Binarization map using Frangi’s filter with small kernel size. (c) Binarization map using Frangi’s filter with large kernel size.*


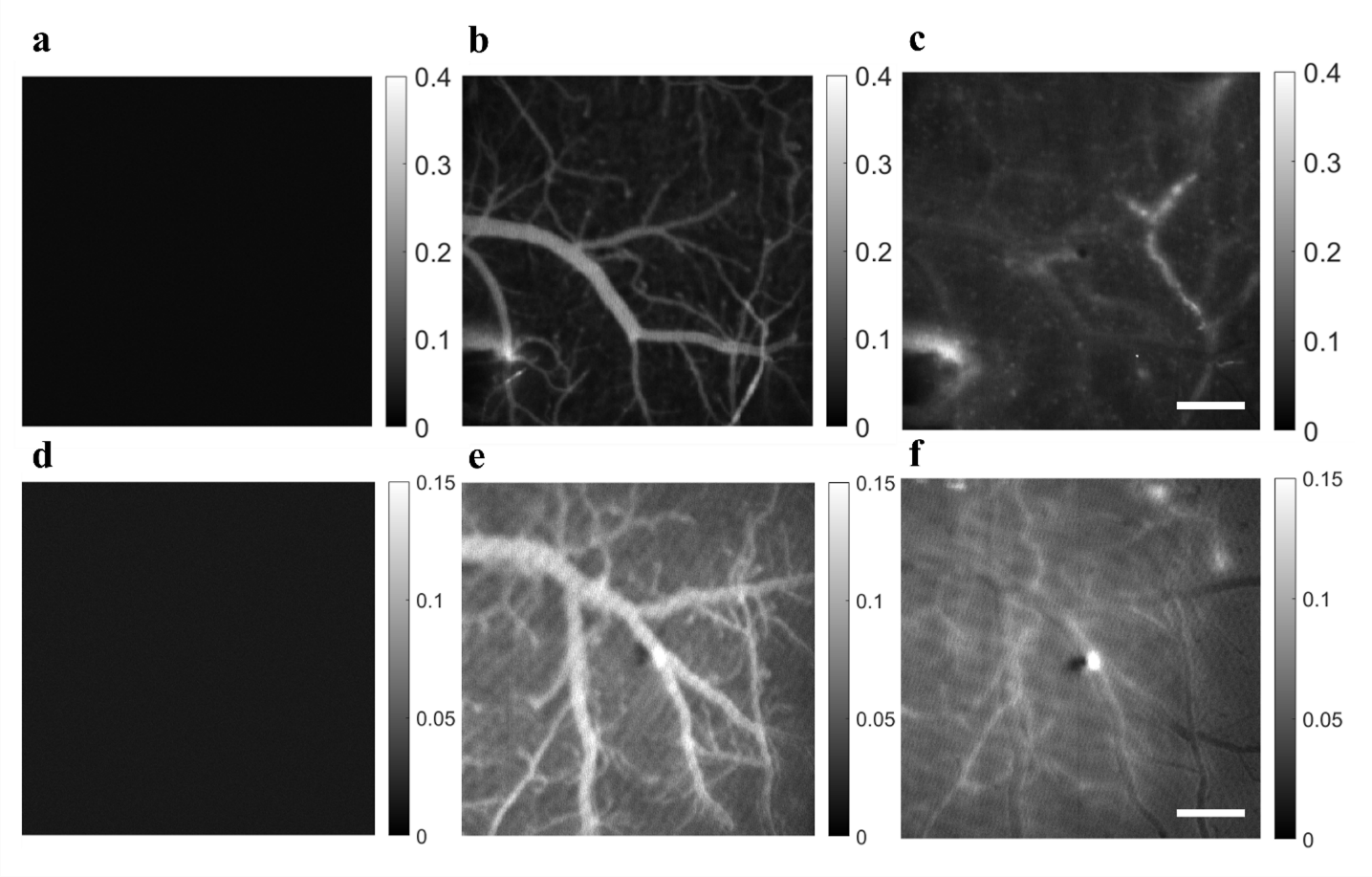


*Supplementary Figure 2. (a)-(c)Fluorescence intensity image of a 10-month-old Alzheimer’s Disease mouse at different time points. (d)-(f) Fluorescence intensity image of a 7-month-old wild type mouse at different time points. (a)(d) Before administration of CRANAD-3; (b)(e) 5 mins after the administration; (c)(f) 1 hour after the administration. Scalebar represents 400 µm*


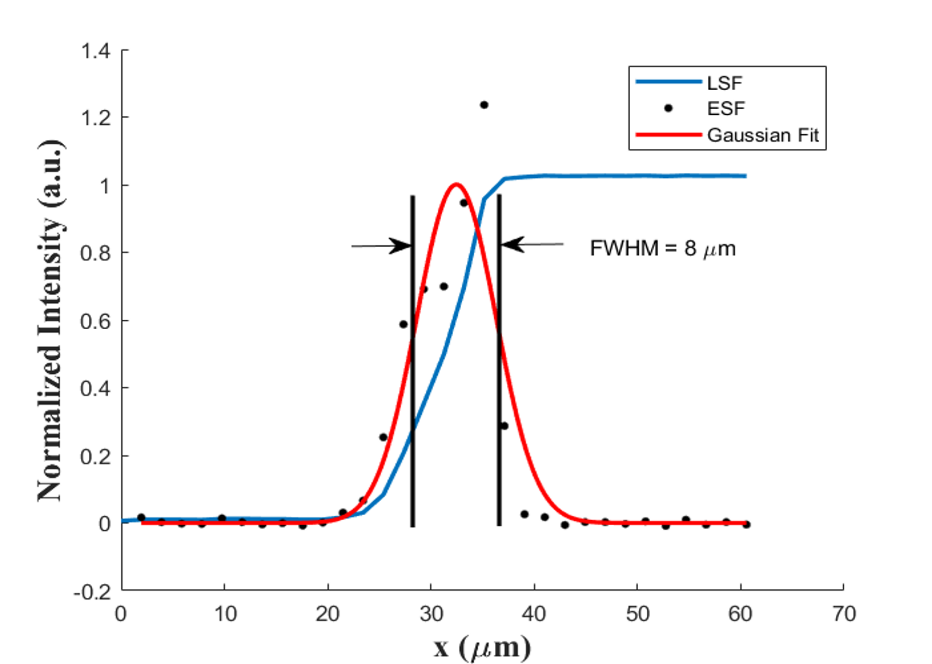


*Supplementary Figure 3. The edge spread function (ESF) and the line spread function (LSF) of the PAM system. The ESF is represented by the intensity profile of a line scanning across the edge of a metal blade.*

*
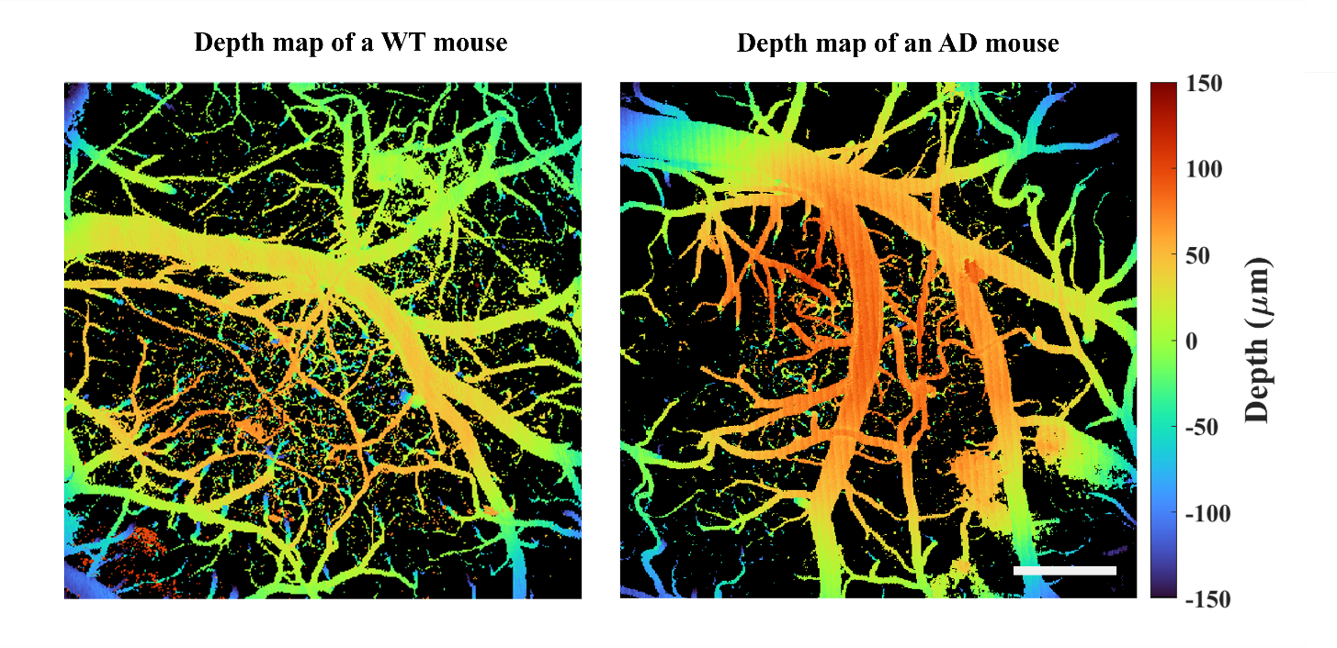
*

*Supplementary Figure 4. Exemplary depth-encoded image from a wild-type mouse and an Alzheimer’s Disease mouse. Depth is calculated based on the speed of sound divided by the arrival time of the signal to the transducer. Scale bar represents 400 μm.*
